# Characteristics of brain computed tomography in dementia with cardiovascular disease and psychological and behavioral symptoms

**DOI:** 10.3389/fneur.2026.1714782

**Published:** 2026-03-04

**Authors:** Qiangqiang Dong, Jian Li, Xinshan Guo, Zhimei Gao, Zhanhui Liu, Deyuan Zhao, Zeqiang Ji

**Affiliations:** Department of Radiology and Nuclear Medicine, The First Hospital of Hebei Medical University, Shijiazhuang, China

**Keywords:** brain CT scan, cardiovascular diseases, correlation, dementia, neuropsychiatric symptoms

## Abstract

**Objective:**

To describe brain computed tomography (CT) features in Alzheimer’s disease (AD) with comorbid cardiovascular diseases (CVDs) and examine associations with behavioral and psychological symptoms (BPSD).

**Methods:**

This single-center, hospital-based observational case-control study (August 2019–May 2021) used consecutive sampling. We enrolled 165 older adults with AD and CVDs (CVD group), 165 older adults with AD without CVDs (AD-only group), and 165 cognitively healthy older adults (healthy controls). All participants underwent non-contrast brain CT at baseline. Qualitative CT findings [cortical atrophy, widened sulci, and medial hippocampal cerebrospinal fluid (CSF) pool widening] and quantitative parameters (lateral split brain width, frontal sulcus width, lateral ventricle width, third ventricle width, forehead index, and caudate nucleus index) were compared across groups. Diagnostic performance for AD (AD groups vs. healthy controls) was evaluated using receiver operating characteristic (ROC) curves and the area under the curve (AUC). BPSD were assessed with the Neuropsychiatric Inventory Questionnaire (NPI-Q), and correlations between NPI-Q scores and CT parameters were analyzed in the CVD group.

**Results:**

Qualitative CT abnormalities were more frequent in both AD groups than in healthy controls (*p* < 0.05) but did not differ between the CVD and AD-only groups (*p* > 0.05). Quantitative CT parameters showed a similar pattern: both AD groups differed from healthy controls (*p* < 0.05), while comparisons between the two AD groups were not significant (*p* > 0.05). The combined diagnostic AUC for AD was 0.881. In the CVD group, higher NPI-Q total scores were associated with decreased lateral split brain width and increased frontal sulcus width, lateral ventricle width, third ventricle width, forehead index, and caudate nucleus index (all *p* < 0.05).

**Conclusion:**

AD participants, with or without CVD comorbidity, showed significant CT abnormalities compared with healthy controls. In AD with CVDs, quantitative CT parameters were associated with BPSD severity.

## 1 Introduction

Alzheimer’s disease (AD) is the leading cause of dementia in older adults and is characterized by progressive cognitive impairment and functional decline. Many individuals with dementia also develop behavioral and psychological symptoms of dementia (BPSD; neuropsychiatric symptoms), including agitation, depression, anxiety, apathy, irritability, sleep disturbance, and related behavioral changes. These symptoms increase caregiver burden and reduce quality of life (1, 2). Epidemiological evidence indicates that the global burden of AD continues to rise, posing substantial challenges for prevention and treatment in China and worldwide (3, 4).

Cardiovascular diseases (CVDs) are common comorbidities in older adults with AD. Vascular risk factors and CVD-related conditions (e.g., hypertension and dyslipidemia) have been associated with cognitive decline and increased dementia risk, and CVD comorbidity may worsen overall health status and prognosis in dementia (5). Clarifying the relationships among CVD comorbidity, brain structural changes, and BPSD in AD may therefore improve clinical assessment and management.

Neuroimaging is central to dementia evaluation. Brain computed tomography (CT) is widely available and relatively low cost, and it can provide clinically useful information on global and regional atrophy patterns and ventricular enlargement. Both qualitative CT features (e.g., cortical atrophy and sulcal widening) and quantitative indices (e.g., ventricle-related measures and the caudate nucleus index) may support dementia assessment and potentially reflect disease severity. However, evidence remains limited on whether CT-derived structural parameters in AD with CVD comorbidity are associated with BPSD severity.

Objectives and hypothesis. This study aimed to: (1) compare qualitative CT features and quantitative CT parameters among AD with CVDs (AD + CVD), AD without CVDs (AD-only), and cognitively healthy older adults; (2) evaluate the diagnostic performance of CT parameters for AD using receiver operating characteristic (ROC) analysis; (3) compare BPSD severity between the two AD groups using the Neuropsychiatric Inventory Questionnaire (NPI-Q); and (4) examine associations between CT parameters and BPSD severity in the AD + CVD group. We hypothesized that participants with AD + CVD would show more pronounced CT abnormalities and greater BPSD burden than those with AD-only, and that greater BPSD severity would be associated with more severe CT-derived atrophy indices.

## 2 Materials and methods

### 2.1 Design

This was a single-center, hospital-based observational case–control study conducted between August 2019 and May 2021. A non-probabilistic consecutive sampling approach was used. Because the study was exploratory and observational, no *a priori* sample size calculation was performed; the final sample reflected the number of eligible participants who completed all required assessments during the study period.

### 2.2 Participants

We enrolled three groups of older adults (age ≥ 60 years): (1) Alzheimer’s disease (AD) with comorbid cardiovascular diseases (CVDs) (AD + CVD group; *n* = 165), (2) AD without CVDs (AD-only group; *n* = 165), and (3) cognitively healthy older adults (healthy controls; *n* = 165). AD was diagnosed according to the 2018 Chinese Guidelines for the Diagnosis and Treatment of Dementia and Cognitive Impairment (Part II: AD) (6), and CVDs were diagnosed based on the Diagnostic Criteria for Cardiovascular Diseases (7). The CVD subtypes included hypertension (33 cases), angina pectoris (40 cases), coronary heart disease (23 cases), hyperlipidemia (29 cases), and atherosclerosis (40 cases).

Inclusion criteria were: age ≥ 60 years; no history of schizophrenia, depression, or other neurological diseases; ability to undergo brain CT examination; and written informed consent provided by patients and their families. Exclusion criteria were: cerebrovascular disease or diabetes; impaired liver or kidney function; malignant tumors; neurological damage from other causes (e.g., intracranial infection or traumatic brain injury); and mixed or unexplained types of dementia.

### 2.3 Institution/setting

The study was conducted at The First Hospital of Hebei Medical University (Shijiazhuang, Hebei, China). Participants with AD (with or without CVDs) were recruited from inpatient/clinical services where older adults were evaluated for cognitive impairment and referred for non-contrast brain CT as part of routine assessment. Healthy controls were recruited from older adults attending routine health examinations at the same hospital during the same period.

### 2.4 Instruments and materials

Brain CT was performed using a 16-slice spiral CT scanner (United Imaging, uCT 510, Shanghai) with a tube voltage of 120 kV, tube current of 260 mA, and slice thickness of 5 mm; the scanning plane followed the orbitomeatal line. Two radiologists with >5 years of experience independently reviewed the images. Qualitative CT findings included cortical atrophy, widened sulci, and widening of the medial hippocampal cerebrospinal fluid (CSF) pool. Quantitative CT parameters included lateral split brain width, frontal sulcus width, lateral ventricle width, third ventricle width, forehead index, and caudate nucleus index.

Behavioral and psychological symptoms of dementia were assessed using the Neuropsychiatric Inventory-Questionnaire (NPI-Q) (8), covering 12 symptom domains in the past month. Each symptom is rated 0 (absent) to 3 (severe) for a total score of 0–36, with higher scores indicating more severe symptoms. Baseline demographic and clinical variables collected included sex, age, body mass index (BMI), smoking history, drinking history, and education level.

### 2.5 Procedure

Once approval was obtained from the Ethics Committee of the First Hospital of Hebei Medical University (Approval No. 2025-178), eligible participants and their families were approached consecutively during the study period, and written informed consent was obtained. Participants were assigned to the AD + CVD, AD-only, or healthy control group based on the diagnostic criteria described above. Baseline demographic and clinical data were collected. Brain CT was performed at baseline (upon admission for AD participants and on the day of the health examination for controls) using the acquisition protocol described above. CT images were independently evaluated by the two radiologists; any discrepancies were resolved by consensus. Behavioral and psychological symptoms were assessed using the NPI-Q via a structured interview with a knowledgeable informant (and/or the participant) regarding symptoms in the past month; the total score ranges from 0 to 36 and was categorized as mild (0–12), moderate (13–24), or severe (25–36). All data were entered into a study database for statistical analysis.

### 2.6 Statistical analysis

Statistical analyses were performed using SPSS (version 22.0). Continuous variables were assessed for normality (Kolmogorov–Smirnov test and visual inspection) and homogeneity of variance (Levene’s test). Normally distributed continuous variables are presented as mean ± standard deviation and were compared using one-way analysis of variance (ANOVA) with *post-hoc* multiple-comparison procedures. Non-normally distributed continuous variables are presented as median (interquartile range) and were compared using the Kruskal–Wallis test with *post-hoc* pairwise comparisons (with adjustment for multiple testing). Categorical variables are presented as counts (percentages) and were compared using the chi-square test (or Fisher’s exact test when appropriate).

Receiver operating characteristic (ROC) curves were constructed to evaluate the diagnostic performance of CT parameters for distinguishing participants with AD (AD-only and AD + CVD groups) from healthy controls; the area under the curve (AUC) with 95% confidence intervals was reported. Associations between NPI-Q total score and CT quantitative parameters within the AD + CVD group were assessed using Pearson correlation when normality assumptions were met, and Spearman rank correlation otherwise. All tests were two-tailed, and *p* < 0.05 was considered statistically significant.

## 3 Results

### 3.1 Comparison of general information and brain CT characteristics among the three groups

**Figure 1:**
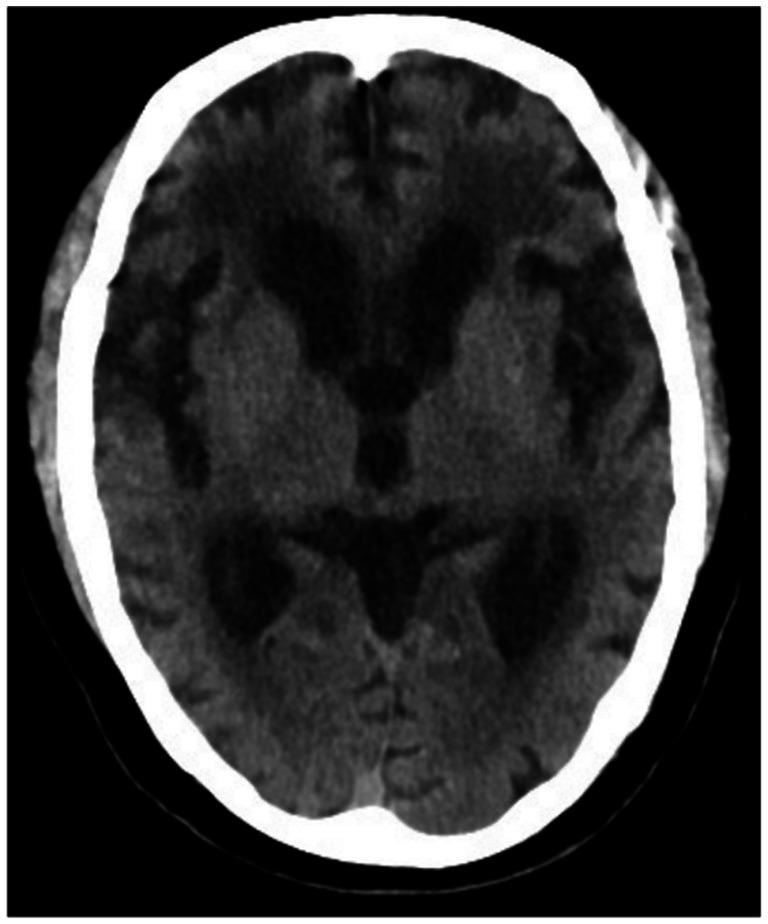
Typical brain CT scan image of the research group. A 63-year-old female patient with dementia and cardiovascular disease presented with significant cortical atrophy, widening of the sulci, and enlargement of the hippocampal medial cerebrospinal fluid pool, along with ischemic white matter changes. Additionally, widening of the lateral sulcus, increased depth and width of the frontal sulcus, and enlargement of both the lateral and third ventricles were observed.

**Figure 2:**
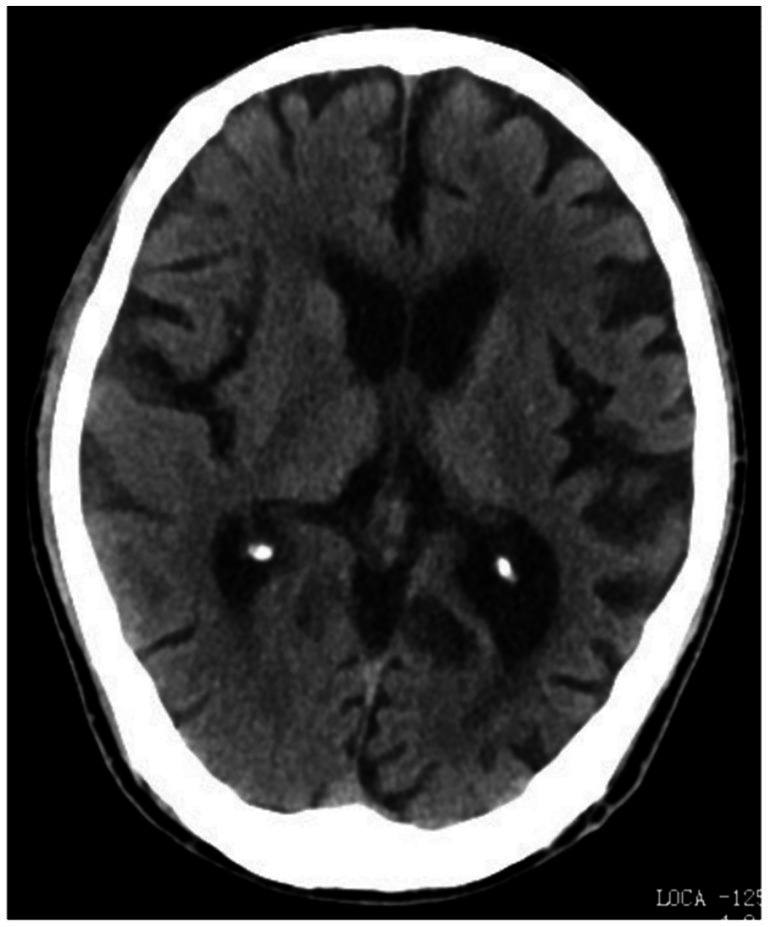
Comparison of typical brain CT scan images of group A. A 63-year-old male patient with pure dementia exhibited noticeable cortical atrophy, widening of the sulci, and enlargement of the hippocampal medial cerebrospinal fluid pool. Additionally, there was widening of the lateral sulcus, increased depth and width of the frontal sulcus, and enlargement of the lateral ventricles.

**Figure 3:**
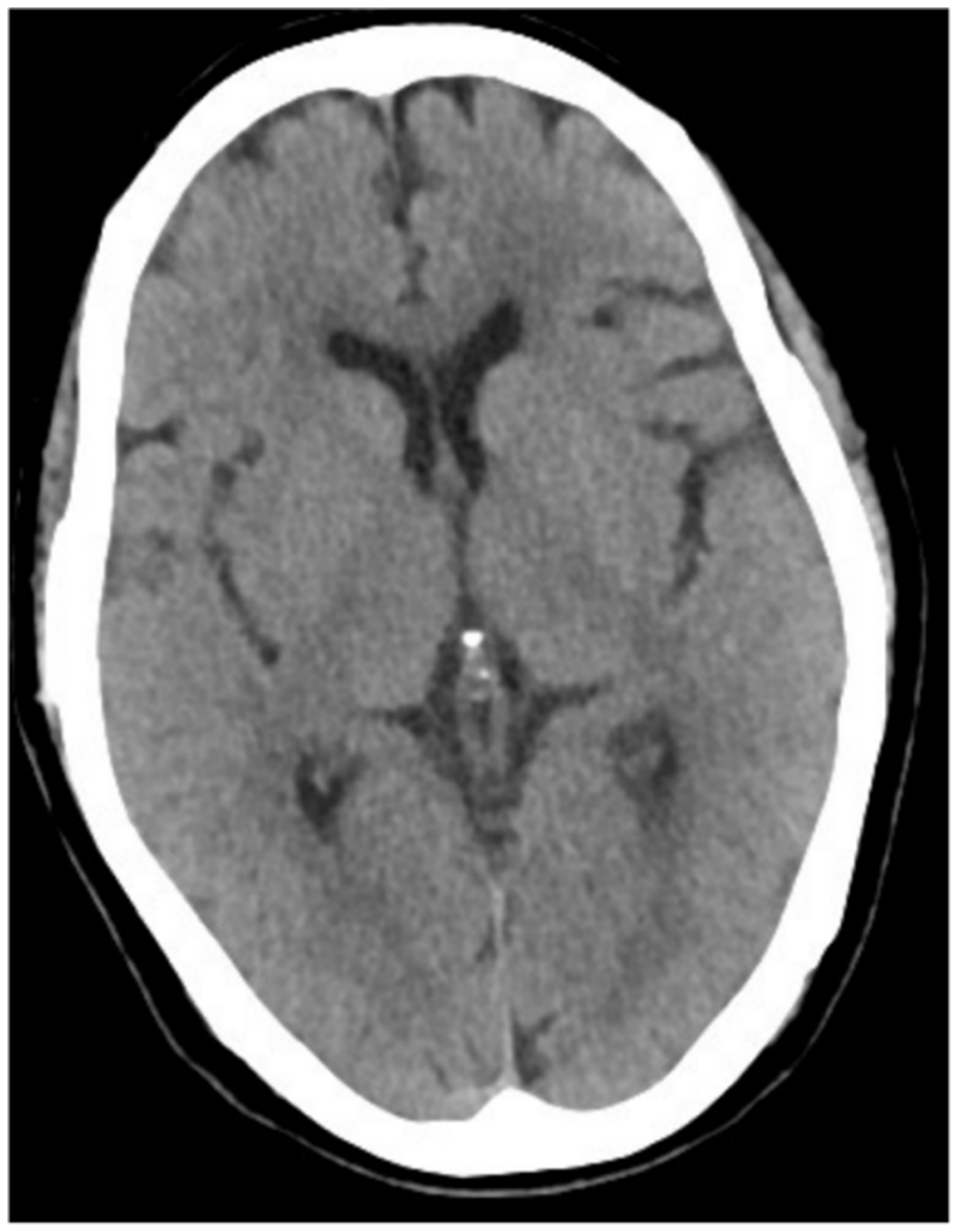
Comparison of typical brain CT scan images of group B. A 64-year-old female, a healthy elderly individual, displayed normal cortical thickness, with symmetrical bilateral lateral ventricles and no enlargement. All cerebral ventricles and sulci were of normal size, with no widening or deepening observed.

There were no significant differences in sex, age, body mass index (BMI), smoking history, drinking history, or educational level among the three groups (*p* > 0.05). The rates of cortical atrophy, widening of cerebral sulci, and enlargement of the medial hippocampal cerebrospinal fluid (CSF) pool were significantly higher in the study group and control group A compared to control group B (*p* < 0.05). While these rates were also higher in the study group than in control group A, the differences were not statistically significant (*p* > 0.05). Detailed comparisons are shown in Table 1, and representative brain CT images are presented in Figures 1–3.

**Table 1:** Comparison of general data and characteristics of brain CT scan in three groups [( $\bar{x}$ ± *s*)/*n*(%)].

Index	Study group (*n* = 165)	Control group A (*n* = 165)	Control group B (*n* = 165)	Statistic (*F*/*χ^2^*/*u*)	*p*-value
Gender (male/female)	73/92	66/99	84/81	4.031	0.133
Age (year)	67.18 ± 3.68	66.85 ± 3.42	66.91 ± 2.95	0.451	0.638
Body mass index (kg/m^2^)	25.13 ± 2.06	24.86 ± 2.23	24.59 ± 2.17	2.591	0.076
Smoking history (yes/no)	65/100	57/108	60/105	0.852	0.653
History of drinking (yes/no)	77/88	68/97	65/100	1.935	0.380
Education				0.636	0.817
Junior high school and below	95(57.58)	90(54.55)	100(60.61)		
High school and technical secondary school	37(22.42)	40(24.24)	35(21.21)		
College degree and above	33(20.00)	35(21.21)	30(18.18)		
Cortical atrophy	139(84.24)^a^	125(75.76)^a^	22(13.33)^a^	202.840	<0.001
Widened sulci	150(90.91)^a^	143(86.67)^a^	34(20.61)^a^	228.740	<0.001
Widened cerebrospinal fluid pool in the medial hippocampus	161(97.58)^a^	152(92.12)^a^	40(24.24)^a^	269.255	<0.001

Compared with the control group B.

a *p*<0.05.

### 3.2 Comparison of brain CT parameters among the three groups

The sulcus width of frontal lobe, lateral ventricular width, third ventricular width, forehead index Value, and caudate nucleus index were significantly higher in both the study group and control group A compared to control group B (*p* < 0.05). Conversely, the lateral split brain width was significantly lower in both the study group and control group A compared to control group B (*p* < 0.05). No statistically significant differences in brain CT parameters were observed between the study group and control group A (*p* > 0.05). These findings are summarized in Table 2.

**Table 2:** Comparison of three groups of brain CT plain scan parameters ( $\bar{x}$ ± *s*).

Index	Study group (*n* = 165)	Control group A (*n* = 165)	Control group B (*n* = 165)	Statistic (*F*)	*p*-value
Lateral split brain width	0.63 ± 0.20^a^	0.65 ± 0.18^a^	1.16 ± 0.31	225.908	<0.001
Sulcus width of frontal lobe	2.81 ± 0.81^a^	2.68 ± 0.73^a^	1.41 ± 0.52	202.932	<0.001
Lateral ventricle width	4.46 ± 0.72^a^	4.39 ± 0.68^a^	3.32 ± 0.45	170.773	<0.001
Third ventricle width	7.12 ± 2.26^a^	7.06 ± 2.13^a^	4.31 ± 0.96	120.729	<0.001
Forehead Index Value	35.91 ± 3.41^a^	35.56 ± 3.36^a^	31.79 ± 2.14	93.939	<0.001
Caudate nucleus index value	18.17 ± 3.25^a^	18.08 ± 3.17^a^	13.24 ± 2.28	152.595	<0.001

Compared with the control group B.

a *p*<0.05.

### 3.3 Diagnostic value of brain CT scan parameters for AD

**Figure 4:**
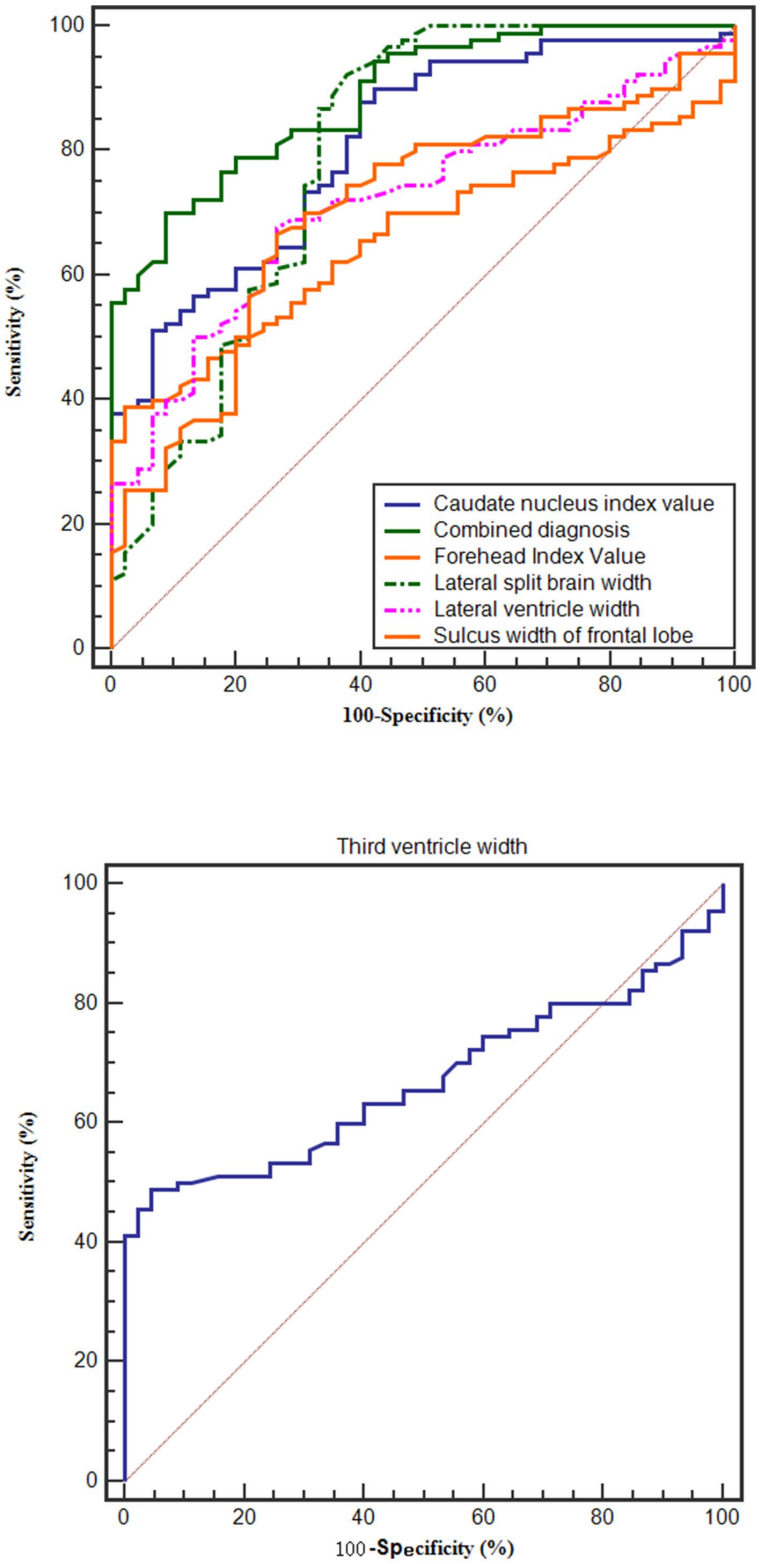
Diagnostic ROC curve.

The brain CT scan parameters were used to diagnose AD by constructing ROC curves with the study group and control group A as positive samples, and control group B as negative samples. The results showed that the AUC for diagnosing AD with the lateral split brain width, sulcus width of frontal lobe, lateral ventricular width, third ventricular width, forehead index Value, and caudate nucleus index were 0.788, 0.752, 0.758, 0.821, 0.745, and 0.789, respectively. The combined diagnostic AUC was the highest at 0.881. These results are presented in Table 3 and Figure 4.

**Table 3:** The value of brain CT plain scan parameters in the diagnosis of AD.

Index	AUC	95% CI	*Z* statistics	Cut-off value	Sensitivity (%)	Specificity (%)	*p*
Lateral split brain width	0.788	0.749 ~ 0.823	12.019	≤0.82	86.36	62.42	<0.001
Sulcus width of frontal lobe	0.752	0.712 ~ 0.790	11.667	>2.55	51.52	90.91	<0.001
Lateral ventricle width	0.758	0.717 ~ 0.795	11.956	>4.03	56.67	85.45	<0.001
Third ventricle width	0.821	0.785 ~ 0.854	17.539	>6.09	66.67	92.12	<0.001
Forehead index value	0.745	0.704 ~ 0.783	11.146	>34.17	58.79	79.39	<0.001
Caudate nucleus index value	0.789	0.751 ~ 0.824	14.495	>16.41	57.27	90.91	<0.001
Combined diagnosis	0.881	0.850 ~ 0.909	25.186	–	71.52	87.27	<0.001

### 3.4 Comparison of NPI-Q scores between the study group and control group A

The NPI-Q score in the study group was 17.61 ± 2.23, while in control group A it was 16.79 ± 2.15. The difference between the two groups was not statistically significant (*t* = 1.776, *p* = 0.079).

### 3.5 Comparison of brain CT scan parameters among patients with different severity levels of neuropsychiatric symptoms in the study group

In the study group, there were 48 patients with mild neuropsychiatric symptoms, 81 with moderate symptoms, and 36 with severe symptoms. As the severity of neuropsychiatric symptoms increased, the lateral split brain width showed a gradual decrease, whereas the sulcus width of frontal lobe, lateral ventricular width, third ventricular width, forehead index value, and caudate nucleus index demonstrated progressive increases (*p* < 0.05). These findings are detailed in Table 4.

**Table 4:** Comparison of brain CT scan parameters of patients with different mental and behavioral symptoms in the study group ( $\bar{x}$ ± *s*).

Index	Mild (*n* = 48)	Moderate (*n* = 81)	Severe (*n* = 36)	Statistic(*F*)	*p*-value	*ηp^2^*
Lateral split brain width	0.77 ± 0.18	0.63 ± 0.16^a^	0.44 ± 0.14^a^^,^^b^	42.630	<0.001	0.345
Sulcus width of frontal lobe	2.17 ± 0.65	2.89 ± 0.71^a^	3.49 ± 0.90^a^^,^^b^	33.691	<0.001	0.294
Lateral ventricle width	3.88 ± 0.59	4.47 ± 0.65^a^	5.21 ± 0.75^a^^,^^b^	42.218	<0.001	0.343
Third ventricle width	4.84 ± 1.75	7.26 ± 2.14^a^	9.84 ± 2.61^a^^,^^b^	55.980	<0.001	0.409
Forehead index value	33.66 ± 2.93	35.97 ± 3.27^a^	38.76 ± 3.83^a^^,^^b^	24.460	0.003	0.232
Caudate nucleus index value	15.30 ± 2.86	18.38 ± 3.17^a^	21.54 ± 3.62^a^^,^^b^	39.727	<0.001	0.329

a *p* < 0.05 compared with mild.

b *p* < 0.05 compared with moderate. Effect size is reported as partial eta-squared (*ηp*^2^).

One-way ANOVA with Bonferroni-adjusted *post-hoc* tests.

Detailed Bonferroni *post-hoc* statistics and Cohen’s *d* are provided in Supplementary Table S1.

### 3.6 Correlation analysis between brain CT scan parameters and neuropsychiatric symptoms

Pearson correlation analysis revealed a negative correlation between lateral split brain width and the NPI-Q score in patients with dementia and cardiovascular diseases. Conversely, sulcus width of frontal lobe, lateral ventricular width, third ventricular width, forehead index Value, and caudate nucleus index were positively correlated with the NPI-Q score (*p* < 0.05). Details are provided in Table 5.

**Table 5:** Correlation analysis of brain CT plain scan parameters and mental behavior symptoms.

Index		Lateral split brain width	Sulcus width of frontal lobe	Lateral ventricle width	Third ventricle width	Forehead index value	Caudate nucleus index value
NPI-Q	*r*	−8.126	8.035	9.827	10.668	6.129	8.416
NPI-Q score	*p*	<0.001	<0.001	<0.001	<0.001	<0.001	<0.001

## 4 Discussion

This study characterized non-contrast CT features in patients with AD and cardiovascular comorbidity and evaluated their clinical relevance to BPSD. Specifically, we compared qualitative and quantitative CT features across AD + CVD, AD-only, and healthy control groups; assessed the diagnostic utility of CT parameters using ROC analysis; compared BPSD severity between AD + CVD and AD-only; and examined associations between CT parameters and BPSD severity within the AD + CVD group.

Dementia is a progressive central nervous system disorder with multifactorial etiology, involving demographic factors, genetic predisposition, and common medical conditions (9, 10). Emerging evidence suggests that CVDs contribute to dementia development and frequently co-occur with dementia, particularly hypertension and related cardiovascular conditions (11, 12). Neuroimaging is integral to dementia diagnosis and management, and CT remains one of the most commonly used modalities in clinical practice because of its accessibility, ease of use, and relatively low cost. CT can depict gross structural abnormalities and supports clinical decision-making in dementia assessment (13).

In our study, qualitative CT findings—including cortical atrophy, sulcal widening, and enlargement of the medial hippocampal cerebrospinal fluid pool—were more frequent in patients with AD than in healthy older adults, consistent with prior reports [e.g., (14)]. These findings support the presence of marked cortical and hippocampal atrophy in AD.

When comparing AD patients with and without cardiovascular comorbidity, qualitative CT abnormalities tended to be more frequent in the AD + CVD group, although group differences were not statistically significant. Some prior studies report that hypertension may impair cerebral autoregulation and increase white matter lesions in dementia (15). The lack of significant differences in our sample may reflect limited statistical power due to the modest sample size.

CT can also support dementia assessment through quantitative indices of brain atrophy, which may facilitate earlier recognition and intervention (16). In this study, we quantified structural parameters including lateral ventricle width, frontal gyrus width, third ventricle width, frontal angle index, and caudate nucleus index. Lateral ventricle width is commonly used to estimate frontotemporal atrophy, frontal gyrus width reflects global cortical atrophy, and the frontal angle index has been linked to myelin-related changes (17, 18). The hippocampus is critical for learning and memory; hippocampal injury or atrophy contributes to cognitive impairment. Indices such as the hippocampal index and ventricular enlargement are considered indirect markers of hippocampal atrophy (19).

Compared with healthy controls, patients with AD showed significantly altered quantitative CT parameters, consistent with frontal–temporal atrophy, ventricular enlargement, and related structural changes. In ROC analysis, CT parameters demonstrated diagnostic value for AD; the combined model yielded an area under the curve (AUC) of 0.881, indicating excellent performance. These results support the potential utility of CT-derived indices as adjunctive markers in dementia workups, particularly in settings where MRI is not readily available.

BPSD are often conceptualized as manifestations of unmet needs in dementia; timely identification and tailored management can mitigate symptom severity (20). In this study, NPI-Q scores tended to be higher in the AD + CVD group, although the difference was not statistically significant. Within the AD + CVD group, increasing BPSD severity was associated with progressively more abnormal CT-derived structural indices: lateral split-brain width decreased, whereas frontal gyrus width, lateral ventricle width, third ventricle width, frontal angle index, and caudate nucleus index increased. These parameters were closely correlated with NPI-Q scores, suggesting that CT-derived measures may help quantify structural changes associated with neuropsychiatric symptom burden in AD patients with cardiovascular comorbidity.

### 4.1 Limitations, future directions, and clinical relevance

This study has several limitations. The single-center, cross-sectional case–control design and hospital-based sampling may limit generalizability and preclude causal inference. Cardiovascular diseases were analyzed as a heterogeneous comorbidity group without stratification by subtype, severity, or treatment/control status, and residual confounding from clinical factors may remain. In addition, non-contrast CT (5-mm slice thickness) is less sensitive than MRI for subtle regional atrophy and small-vessel disease markers; BPSD were assessed using the informant-based NPI-Q, which is susceptible to reporting and recall bias.

Future research should use multicenter, prospective longitudinal designs with stratification by CVD burden/subtype, apply multivariable (or propensity-based) adjustment, and incorporate MRI markers and/or AD biomarkers to validate and refine these findings. Clinically, our results suggest that readily available CT-derived quantitative indices may complement routine dementia workups and help identify AD patients with CVD comorbidity who are at higher risk for more severe BPSD, supporting earlier behavioral management and caregiver support, particularly where MRI access is limited.

## 5 Conclusion

Based on the results found and the scientific literature reviewed, we can conclude: (1) Compared with cognitively healthy older adults, participants with Alzheimer’s disease, with or without cardiovascular comorbidity, exhibit more frequent qualitative CT abnormalities and more pronounced quantitative CT-derived indices of atrophy and ventricular enlargement. (2) Quantitative brain CT parameters demonstrate useful diagnostic value for distinguishing Alzheimer’s disease from healthy aging. (3) Behavioral and psychological symptoms of dementia are comparable between Alzheimer’s disease participants with and without cardiovascular diseases in this sample. (4) Within the Alzheimer’s disease with cardiovascular diseases group, greater BPSD severity is associated with more pronounced CT-derived structural changes, including reduced lateral split brain width and increased indices reflecting sulcal widening and ventricular enlargement. Overall, brain CT provides accessible structural markers that complement dementia assessment and may help identify patients with cardiovascular comorbidity who are more likely to present with a higher neuropsychiatric symptom burden.

## Data Availability

The original contributions presented in the study are included in the article/Supplementary material, further inquiries can be directed to the corresponding author.
