# Supplementary material for: Characteristics of brain computed tomography in dementia with cardiovascular disease and psychological and behavioral symptoms
Source: Front Neurol. 2026 Mar 4;17:1714782. doi: 10.3389/fneur.2026.1714782 (PMC12995796; doi:10.3389/fneur.2026.1714782)
Supplement: Supplementary file 1 [file Table_1.docx]

**Supplementary Table S1. Bonferroni-adjusted post-hoc pairwise comparisons for Table 4**

| Variable | Comparison | Mean diff (A-B) | t | df | Bonferroni p | Cohen d (A-B) |
| --- | --- | --- | --- | --- | --- | --- |
| Lateral split brain width | Mild vs Moderate | 0.14 | 4.58 | 127 | <0.001 | 0.83 |
| Lateral split brain width | Mild vs Severe | 0.33 | 9.12 | 82 | <0.001 | 2.01 |
| Lateral split brain width | Moderate vs Severe | 0.19 | 6.15 | 115 | <0.001 | 1.23 |
| Sulcus width of frontal lobe | Mild vs Moderate | -0.72 | -5.74 | 127 | <0.001 | -1.05 |
| Sulcus width of frontal lobe | Mild vs Severe | -1.32 | -7.81 | 82 | <0.001 | -1.72 |
| Sulcus width of frontal lobe | Moderate vs Severe | -0.60 | -3.88 | 115 | <0.001 | -0.78 |
| Lateral ventricle width | Mild vs Moderate | -0.59 | -5.15 | 127 | <0.001 | -0.94 |
| Lateral ventricle width | Mild vs Severe | -1.33 | -9.10 | 82 | <0.001 | -2.01 |
| Lateral ventricle width | Moderate vs Severe | -0.74 | -5.42 | 115 | <0.001 | -1.09 |
| Third ventricle width | Mild vs Moderate | -2.42 | -6.63 | 127 | <0.001 | -1.21 |
| Third ventricle width | Mild vs Severe | -5.00 | -10.50 | 82 | <0.001 | -2.32 |
| Third ventricle width | Moderate vs Severe | -2.58 | -5.62 | 115 | <0.001 | -1.13 |
| Forehead index value | Mild vs Moderate | -2.31 | -4.03 | 127 | <0.001 | -0.73 |
| Forehead index value | Mild vs Severe | -5.10 | -6.92 | 82 | <0.001 | -1.53 |
| Forehead index value | Moderate vs Severe | -2.79 | -4.04 | 115 | <0.001 | -0.81 |
| Caudate nucleus index value | Mild vs Moderate | -3.08 | -5.53 | 127 | <0.001 | -1.01 |
| Caudate nucleus index value | Mild vs Severe | -6.24 | -8.83 | 82 | <0.001 | -1.95 |
| Caudate nucleus index value | Moderate vs Severe | -3.16 | -4.76 | 115 | <0.001 | -0.95 |

Caption: Detailed post-hoc statistics are reported with Bonferroni-adjusted P values. Effect sizes for pairwise comparisons are provided as Cohen’s d.
